# Molecular analysis of meso- and thermophilic microbiota associated with anaerobic biowaste degradation

**DOI:** 10.1186/1471-2180-12-121

**Published:** 2012-06-22

**Authors:** Jarmo Ritari, Kaisa Koskinen, Jenni Hultman, Jukka M Kurola, Maritta Kymäläinen, Martin Romantschuk, Lars Paulin, Petri Auvinen

**Affiliations:** 1 Institute of Biotechnology, University of Helsinki, Viikinkaari 4, 00790, Helsinki, Finland; 2 Department of Environmental Sciences, University of Helsinki, Niemenkatu 73 C, 15140, Lahti, Finland; 3 HAMK University of Applied Sciences, P.O.Box 230, 13101, Hämeenlinna, Finland; 4 Current address: Department of Food Hygiene and Environmental Health, Faculty of Veterinary Medicine, University of Helsinki, Agnes Sjöbergin katu 2, 00790, Helsinki, Finland

## Abstract

**Background:**

Microbial anaerobic digestion (AD) is used as a waste treatment process to degrade complex organic compounds into methane. The archaeal and bacterial taxa involved in AD are well known, whereas composition of the fungal community in the process has been less studied. The present study aimed to reveal the composition of archaeal, bacterial and fungal communities in response to increasing organic loading in mesophilic and thermophilic AD processes by applying 454 amplicon sequencing technology. Furthermore, a DNA microarray method was evaluated in order to develop a tool for monitoring the microbiological status of AD.

**Results:**

The 454 sequencing showed that the diversity and number of bacterial taxa decreased with increasing organic load, while archaeal i.e. methanogenic taxa remained more constant. The number and diversity of fungal taxa increased during the process and varied less in composition with process temperature than bacterial and archaeal taxa, even though the fungal diversity increased with temperature as well. Evaluation of the microarray using AD sample DNA showed correlation of signal intensities with sequence read numbers of corresponding target groups. The sensitivity of the test was found to be about 1%.

**Conclusions:**

The fungal community survives in anoxic conditions and grows with increasing organic loading, suggesting that Fungi may contribute to the digestion by metabolising organic nutrients for bacterial and methanogenic groups. The microarray proof of principle tests suggest that the method has the potential for semiquantitative detection of target microbial groups given that comprehensive sequence data is available for probe design.

## Background

Anaerobic digestion (AD) is a microbiological process where organic material is degraded by numerous different groups of microorganisms [1]. The AD process consists of three main steps. First, the complex organic material is hydrolysed. Then, in acidogenesis and acetogenesis, the generated less complex substrates are converted into acetate, hydrogen and carbon dioxide from which methane is finally produced in methanogenesis [2]. At least four different trophic groups are essential for methanogenic degradation: 1) fermentative heterotrophs decompose organic materials such as proteins, lipids and carbohydrates, 2) proton-reducing H_2_-producing heterotrophic syntrophs are involved in degradation of small molecules like fatty acids and ketones, and, 3) H_2_-utilising and 4) aceticlastic methanogenic archaea produce the methane [3].

Biowaste used as a substrate for AD contains different organic materials from food crop residues to waste originating from industrial processing. The microbial community present in the AD process is largely determined by the substrate composition [1] and reactor design as well as operating conditions [4]. One of the important operating conditions is temperature which affects the microbial diversity of the AD process drastically: in mesophilic (temperature about 35 °C) conditions, the species richness and the number of different microbial phyla appear to be higher and the species composition very different compared to thermophilic (temperature about 55 - 60 °C) conditions. Nevertheless, the AD reactor performance is relatively similar in both temperatures, except for the more efficient degradation of some specific organic compounds and the presence of pathogens at higher temperatures [5,6]. However, a temperature exceeding 64 °C has been observed to cause acetic acid build-up and process failure leading to diminished methane production [7].

While the abundance and distribution of Bacteria and Archaea in AD processes are well characterised [4,6,8-11], the analysis of Fungi present in the process has been largely overlooked. Fungi are typically discussed in studies of AD only as a potential source of pathogens like *Aspergillus*[12] without considering their possible role in digestion. It has been reported that the total number of fungal colony forming units is not reduced during the AD process in either mesophilic or thermophilic reactors, but still the number of fungal genera is significantly decreased [12]. However, there are known aerobic microbial e.g. fungal groups present in anaerobic digesters originating from the substrate [1]. The aerobic groups stay viable and can therefore form colonies when plated, which may cause biased results when using culturing methods to measure the microbial abundance and distribution [1]. Hence, analysis of phylogenetic marker gene sequences would provide a more reliable characterisation of the composition of microbial communities in the AD process. Our aim in this study was to reveal the molecular phylogenetic structure of bacterial and archaeal and also the fungal communities in AD process operating at different temperatures and organic loads using 454-pyrosequencing. Furthermore, we utilised the 454 sequence data to evaluate a DNA microarray method for monitoring the microbiota in the AD process. Such DNA microarray technology could enable a rapid, almost on-line monitoring of the microbial situation in the process and the digestate reject waters, when needed. Hygienisation of solid and liquid products of the process could also be confirmed without causing delays to the further handling of the products.

## Methods

### Anaerobic reactor and test runs

**Figure 1:**
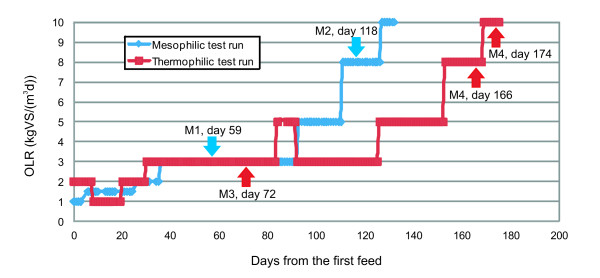
**Organic loading as a function of time in meso- and thermophilic AD reactors.** The arrows point the sampling times (M1, M2, M3 and M4).

The pilot scale anaerobic digestion (AD) reactor has been previously described in detail [13]. In brief, the AD reactor was a completely stirred tank reactor (200 L; operating volume of 150 L) which was fed semi-continuously (once per day) with a mixture of biowaste and sewage sludge (30% and 70% of total wet weight, respectively). The reactor was first run in a mesophilic temperature range of 35 - 38 °C, and later in a thermophilic range of 52 - 56 °C. The organic loading rate (OLR) was increased stepwise from 1 to 10 kgVS m^-3^d^-1^ (kg volatile solids per m^3^ reactor volume and day) (Figure 1). At the same time, HRT (hydraulic retention time) was decreased stepwise from 58 days to 8 days. The selected AD process parameters of the test runs are presented in Tables 1 and 2. The total solids (TS%) were determined by drying samples at 105 °C. The volatile solids (VS%) were determined by volatilizing the organic matter in a muffle oven for 2 h at 550 °C. The alkalinity and total amount of volatile fatty acids (VFA) were determined by a titration method [14]. First the sample was titrated to pH 4 (alkalinity), then to pH 3.3 at which the sample was boiled to release CO_2_. The amount of VFAs was determined by back titration with NaOH from pH 4 to pH 7.

**Table 1:** Physical and chemical process parameters of the pilot AD reactor prior to samplings for DNA extraction during the mesophilic and thermophilc test runs

	**Mesophilic Low load, M1**	**Mesophilic High load, M2**	**Thermophilic Low load, M3**	**Thermophilic High load, M4**
**Process parameters**				
OLR^*)^, kgVS m^-3^d^-1^	3	8	3	8-10
HRT ^**)^, days	22	10	23	9
Temperature, °C	36-37	36-37	54-55	54-55
pH	7.4-7.5	7.4-7.5	8.0-8.1	8.0-8.1
NH_4_-N, g liter^-1^	1.1-1.2	1.2-1.3	1.6-1.7	1.0-1.1
Alkalinity, mgCaCO_3_ liter^-1^	5400 - 6000	6300 - 6700	6200 - 6700	4900 - 5300
VFA^***)^, mg liter^-1^	110 - 160	200 - 340	480 - 590	350 - 600
TS, %	3.1 – 3.2	4 – 4.5	3.2 – 3.3	3.7 – 4.2
VS, %	1.6 – 1.8	2.4 – 2.9	2.0 – 2.1	2.3 – 2.7
TS-reduction ^****)^, %	61 - 62	60 - 62	60 - 62	55 – 60
VS-reduction, %	72 - 74	66 - 69	70 - 71	64 - 70
**Feed characteristics**				
TS, %				
Biowaste (BW)	14.9 – 24.6	29 – 32.2	26.7	29.9 – 21.1
Sewage sludge (SS)	4.1 – 4.2	3.1 – 4.8	3.3 – 4.1	4.5 – 6.0
BW and SS mixture	8.6 – 10.3	11.8 – 13.0	10.7 – 10.9	9.5 – 10.6
VS, %				
Biowaste (BW)	14.3 – 21.6	21.8 – 26.2	24.6	18 – 19.1
Sewage sludge (SS)	2.7 – 3.6	1.8 – 3.2	1.9 – 2.6	2.8 – 3.7
BW and SS mixture	6.2 – 8.4	7.9 – 8.8	8.7 – 9.2	7.4 – 8.0

^*)^ OLR, Organic Loading Rate. For load increase steps and times, see Figure 1.

^**)^ HRT, Hydraulic Retention Time.

^***)^ VFA, total Volatile Fatty Acids.

^****)^ Reduction = [(TS_feed,in_-TS_digestate, out_)/TS_feed,in_] x 100%.

**Table 2:** **Production of biogas and concentrations of methane and selected trace gases from the pilot AD reactor at organic loads of 3 (M1, M3) and 5–8 (M2, M4) kgVS m**^**-3**^

**Parameter**	Mesophilic Low load, **M1**	Mesophilic High load, **M2**	Thermophilic Low load, **M3**	Thermophilic High load, **M4**
Biogas^*)^ Ndm^3^/kgVS_fed_	646 +/− 47	586 +/− 30	632 +/− 76	496 +/− 71
Methane (%, min-max)	52.3 – 66.0	46.0 – 70,9	51.7 – 68.0	nd
*Trace gases*				
Ammonia, NH_3_ (ppm)	< 3	< 3	83	38
H_2_S (ppm)	< 0.1	< 0.1	nd	< 10
DMS (ppm)	< 0.2	< 0.2	nd	< 5
EtOH (ppm)	10	125	2380	2230

^*)^ average biogas production and standard deviations based on a daily and weekly production amount (liters) and feed (kgVS) at each sampling OLR period. The values are normalized for 273 K.

### Sampling protocol and DNA extraction

Sampling for DNA isolation was done in transient AD reactor conditions, i.e. at the load-increasing points: from 2 to 3 kg VS m^-3^d^-1^, and from 5 to 8 kg VS m^-3^d^-^ both in the mesophilic (M1 and M2) and thermophilic (M3 and M4) runs (Table 3). HRT values for each sampling are given in Table 1. The sample volume of the AD reactor’s digested sludge was 1 mL. Total DNA was extracted from the whole volume (4 x 250 mg) of the samples with FastDNA Spin Kit for Soil according to manufacturer’s instructions (MP Biomedicals, France). Extracted DNA was visualised in agarose gel and the concentration of DNA was measured with NanoDrop ND-1000 spectrophotometer (Thermo Fisher Scientific, Waltham, MA, USA). Prior to use, DNA was stored at −20 °C.

**Table 3:** Numbers and diversity of bacterial, archaeal and fungal sequences

**Group**	Load^*)^	**Reactor type**	**Sample name**	**Number of seq.**	Average read length^**)^	**Observed OTUs**	**Diversity (Shannon)**	**Diversity (Simpson)**	**Richness (Chao1)**	**Richness (Ace)**
Bact.	3	meso	M1	5775	151	610	4,11	0,09	1304	2044
	8	meso	M2	4531	151	483	4,43	0,04	1171	1631
	3	thermo	M3	2056	142	444	4,68	0,05	1065	2070
	8	thermo	M4	5083	146	438	3,87	0,07	1127	1827
Arch.	3	meso	M1	7926	104	135	2,33	0,17	318	510
	8	meso	M2	5593	109	109	1,85	0,33	227	339
	3	thermo	M3	5521	106	95	1,02	0,56	227	375
	8	thermo	M4	10573	107	167	1,66	0,34	387	565
Fungi	3	meso	M1	2850	147	456	4,43	0,06	1068	1609
	8	meso	M2	8714	233	1602	5,57	0,03	3192	4485
	3	thermo	M3	8460	209	1386	5,12	0,05	2617	4304
	8	thermo	M4	16893	220	2162	5,22	0,06	3393	4516

^*)^ kg VS m^-3^.

^**)^ after removing adapters and primers.

### 454 sequencing

The PCR amplification of the sample DNA was conducted with MJ Research PTC-225 thermal cycler (Global Medical Instrumentation) in two stages. First, we amplified the DNA with universal bacterial, archaeal and fungal primers in following conditions: initial denaturation at 94 °C for 5 min, 20 cycles of 94 °C for 30 s, 60 °C for 30 s and 72 °C for 2 min, and a final extension for 5 min with bacterial and archaeal primers (Table 4). With fungal primers the applied annealing temperature was 55 °C. In the first round we used eight replicate reactions per sample and pooled and purified the reactions before the second round. In the second round, the amplification was completed with 10 additional cycles with sample-specific barcode sequences and A- and B-adapters attached to the primers. Each sample was amplified in three replicates. The amount of template varied between 200 ng and 700 ng per reaction (volume 50 μl) depending on sample and primers. The PCR amplifications were carried out in the first round with Phusion (Finnzymes, Espoo, Finland) (Bacteria) and Biotools (Biotools, Madrid, Spain) (Archaea and Fungi), and in the second round with Truestart (Fermentas, Lithauen) DNA polymerases. After the amplifications, the replicates were pooled and the PCR-products were processed as described previously [15]. The sequencing was carried out at the Institute of Biotechnology (Helsinki, Finland) using the 454 GS FLX protocol, yielding read length of about 250 bp (454 Life Sciences, Roche Diagnostics, CT, USA).

**Table 4:** PCR primers used for amplicon sequencing in this study

**Primer**	**Direction**	**Sequence**	**Reference**
Ar344f	forward	ACGGGGCGCAGCAGGCGCGA	[16]
518	reverse	ATTACCGCGGCGGCTG	modified from [17]
CREN512	reverse	CGGCGGCTGACACCAG	[18]
341f	forward	CCTACGGGAGGCAGCAG	[19]
D'	reverse	GTATTACCGCGGCTGCTG	[20]
5.8af	forward	GTGAATCATCGAGTTCTTGAAC	modified from [21]
5.8bf	forward	GTGAATCATCAAATCTTTGAAC	modified from [21]
5.8cf	forward	GTGAATCATCGAGTCTTTGAAC	modified from [21]
5.8df	forward	GTGAATCATCAGTTTTTGAAC	modified from [21]
5.8ef	forward	GCGAATCATCGAATTCTCGAAC	modified from [21]
ITS4	reverse	TCCTCCGCTTATTGATATGC	[22]

### Sequence data processing

**Figure 2:**
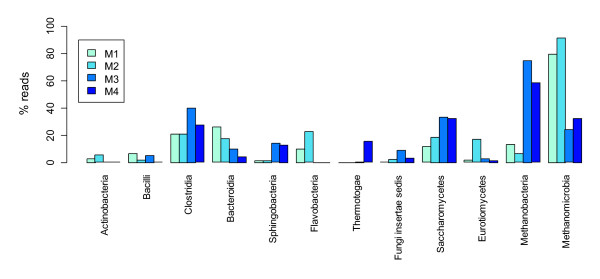
**Overview of microbial diversity in AD samples.** Barplots showing relative sequence numbers of most common microbial groups in samples M1, M2, M3 and M4.

The sequence data was sorted into libraries according to sample-specific barcode sequences using Ribosomal Database Project’s Pyrosequencing pipeline Initial Process [23] and thereafter the tag and the primer sequences were removed. We also left out sequence reads less than 100 bp in length, or with one or more ambiguous nucleotides (N) in order to use only good quality sequences in further analysis [24]. The sequences that passed the initial quality control were analysed with Mothur [25]. Bacterial and archaeal sequences were aligned to SILVA alignment database [26]. Aligned sequences were preclustered, distance matrices were prepared and the sequences were clustered to operational taxonomic units (OTUs) using average neighbor algorithm. Rarefaction curves ( Additional file 1) and ACE [27] and Chao1 [28] indices (Table 3) were calculated to estimate the community richness, and Simpson and Shannon indices [29] were used in assessing the diversity present in samples. We also calculated Venn diagrams and dendrograms describing the shared OTUs within samples and similarity between the structures of communities, respectively. The dendrograms were constructed using the Yue & Clayton similarity value, θ_YC_[30]. Fungal sequences were aligned and distance matrix was prepared using Mothur *pairwise.seqs* command. Clustering and other downstream analyses were carried out as with Bacteria and Archaea. Taxonomic affiliations were determined with BLAST [31] and Megan [32]: sequence reads were queried against the NCBI nucleotide database (*nr/nt*) [33] and the results were analysed using Megan. Fungal sequences affiliated to Plantae or Animalia were removed from the dataset. We applied Ribosomal Database Project’s Classifier [34] to determine the bacterial and archaeal groups present in samples. The sequences have been deposited in the Sequence Read Archive (SRA) at EBI with study accession number ERP000976. The most abundant microbial groups are presented in Figure 2.

### Statistical methods

Redundancy analysis (RDA) ordination technique [35,36] was used to explore the relationships between microbial community composition and variation in physical and chemical parameters. Microbial composition data from both sequencing and microarray were used as dependent variables and six selected physico-chemical parameters as constraints. Only the 12 most abundant microbial classes from sequencing and 12 strongest microarray probes were included in the analysis. Correlation coefficients were used as inertia in the model and plotting. Three different constraining variables were used per analysis because the number of constraining variables is restricted to n-1 (n referring to the number of observations; here M1-M4). Analyses were done using R-software package *vegan* v. 1.17-12 [37].

### Ligation probe design

**Figure 3:**
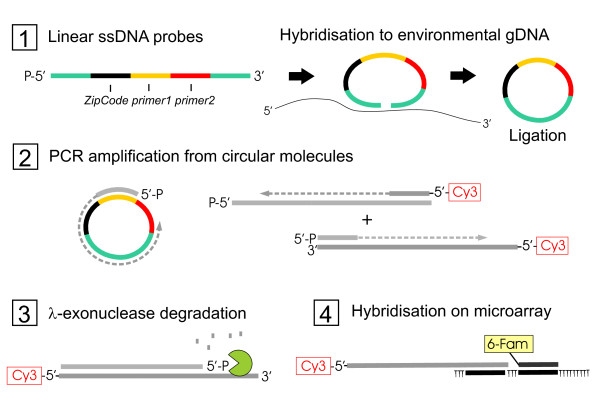
**Schematic figure presenting the principle of the microarray technique.** (**1**.) A linear ssDNA probe containing target recognition sequences at 5’ and 3’ termini is hybridised to environmental gDNA. The probe is ligated into a circular molecule if a complementary target sequence is present. (**2**.) Circular probe is PCR amplified with 5’ phosphorylated forward and 5’ Cy3 labeled reverse primer and (**3**.) thereafter the phosphorylated strand is degraded. (**4**.) The Cy3-labeled products are hybridised on a microarray harbouring complementary ZipCode sequences and a common control probe sequence. Control probe carries a 6-Fam label.

We designed a set of probes consisting of sequences matching to common fungal and bacterial phylotypes (42 probes) and another set that matched OTUs from AD amplicon data (47 probes) ( Additional file 2). The design of ligation probes was based on identification of target-specific nucleotide positions by using sequence alignments and NCBI's Primer-BLAST. First, for those target reads that matched with at least 94% similarity to a full length 16 S rRNA gene in NCBI database, the corresponding 16 S sequences were collected and incorporated into a Greengenes prokaryote 16 S reference database [38]. The minimum length cutoff in the Greengenes database was 1250 bp. A second alignment was constructed of the short pyrosequencing reads representing OTUs. For both alignments, an algorithm that screens for single nucleotide differences was implemented in R-software [39] using Biostrings package [40]. If a specific nucleotide position was identified for a given target sequence, the 3' end of discriminating ligation probe was set to match that position. If no such site was found, Primer-BLAST at the NCBI website was employed to find probe candidates for that target sequence. In Primer-BLAST, the *nr/nt* database was used as reference and primer stringency settings included at least two non-target mismatches in the last four nucleotides in the 3' end. Finally, the T_m_s of selected probes were set to 60 °C and 64 °C for the discriminating and common parts, respectively, using thermodynamic nearest neighbour calculation in Oligocalc software [41]. A schematic of the technique is presented in Figure 3.

### Probe library preparation

The custom oligo library was synthesised by Agilent (Santa Clara, CA) at 10 pmol scale. The dried oligo library, containing 70 fmol of each probe, was dissolved into 70 μl of water and aliquoted to 7 X 10 μl. An aliquot was phosphorylated in a reaction containing 1X PNK buffer A (Fermentas,Lithauen), 0.5 mM ATP and 1 μl of PNK (Fermentas, Lithauen) in a 20 μl volume. The reaction was incubated at 37 °C for 45 min followed by inactivation at 65 °C for 10 min. 30 μl of 0.1X TE buffer was added for final volume of 50 μl and concentration of 400 amol/μl/probe.

### Template fill-in

In order to validate the probes, we designed 96 oligonucleotide templates each consisting of two partially overlapping 50-mer parts. To produce 80-mer double stranded templates from the two oligos, a fill-in reaction containing 1X TrueStart buffer (Fermentas,Lithauen), 1.33 mM MgCl_2_ (Fermentas, Lithauen), 200 μM of each dNTP, 1.6 U of TrueStart Taq DNA polymerase (Fermentas, Lithauen) and 10 μM of both oligos in a 20 μl volume was performed. The program consisted of activation step at 95 °C for 3 min and 5 cycles of denaturation at 95 °C for 30 s, annealing at 55 °C for 30 s and extension at 72 °C for 15 s. Final extension was 15 min at 72 °C. Template oligo sequences are listed in Additional file 3. Ninety-six templates were divided into four pools and each pool was tested separately with all of the probes on the microarray.

### Ligation reaction

Ligation reactions were carried out in a 10 μl volume containing 1X Pfu ligase buffer (Agilent Technologies, Santa Clara, CA, USA), herring sperm DNA (Sigma-Aldrich, Steinheim, Germany), 30 mM tetramethylammonium chloride (TMAC; Sigma-Aldrich, Steinheim, Germany), about 200 ng of environmental template DNA, 400 amol of each probe and 2 U of Pfu ligase (Agilent Technologies, Santa Clara, CA, USA). The reaction was cycled for 20 rounds at 94 °C for 30 s and at 56 °C for 8 min in a thermal cycler (MJ Research, MA, USA).

### PCR from ligated probes

The PCR reaction mixture for amplification of circularised ligation products contained 1X Paq HS buffer (Agilent Technologies, Santa Clara, CA, USA), 200 μM of each dNTP, 0.5 μM forward primer (5'-Cy3-CGACGTTGTAAAACGACGGCCAGT-3'), 0.5 μM reverse primer (5'-phosphate-TTTCACACAGGAAACAGCTATGAC-3'), 2.5 U of Paq5000 DNA polymerase (Agilent Technologies, Santa Clara, CA, USA) and 10 μl of ligation reaction in a final volume of 30 μl. The PCR program consisted of activation step at 95 °C for 3 min and 35 cycles of denaturation at 95 °C for 20 s, annealing at 58 °C for 14 s and extension at 72 °C for 5 s. The PCRs were done in Arktik thermal cycler (Finnzymes, Espoo, Finland) with block-mode temperature control using manufacturer's PCR tubes.

### Microarrays

The microarray experiments were performed on Arrayit or Agilent microarray platforms. The 16 compartment slides purchased from Arrayit (Sunnyvale, CA, USA) were designed and used as described previously [42]. Briefly, for hybridisation to Arrayit microarrays, a mixture containing 20 μl of PCR/lambda exonuclease reaction, 5X SSC, 20 μg of herring sperm DNA (Sigma-Aldrich, Steinheim, Germany) and 5 pmol of control oligo in a final volume of 60 μl was applied to each subarray according to manufacturer's instructions. The hybridisation was carried out in the dark at 55 °C for 2 h. After hybridisation, the microarray was washed for 3X15 min in 0.1X SSC, 0.1% SDS and briefly with water. Finally, the slide was air dried. The high-density custom oligo microarrays were manufactured by Agilent (Santa Clara, CA, USA) in 8 X 15 K format. Each of eight subarrays contained 1500 cZipCode oligos in ten replicates. Hybridisation to Agilent microarrays was performed according to manufacturer's instructions. Briefly, hybridisation mixture containing 1X GEx hybridisation buffer (Agilent Technologies, Cedar Creek, TX, USA), 1X GEx blocking reagent (Agilent Technologies, Cedar Creek, TX, USA), 18 μl of PCR/lambda reaction and 5 pmol of control oligo was applied on each subarray and hybridised for 17 h in the dark at 65 °C at 10 rpm rotation. The slide was washed with Gene Expression wash buffer 1 (Agilent Technologies, Wilmington, DE, USA) for 1 min at RT and Gene Expression wash buffer 2 (Agilent Technologies, Wilmington, DE, USA) for 1 min at 37 °C. 10% Triton X-102 was added to both washing solutions to final concentration of 0.005%. The fluorescent signals were detected at 5 or 10 μm resolution using a GenePix Autoloader 4200AL laser scanning system with green laser for Cy3 dye (ex 543 nm/em 570 nm) and blue laser for 6-FAM (ex 488 nm/em 495 nm). The laser power was set at 100% and the photomultiplier (PMT) tube was adjusted according to the intesity of the signal. GenePix program version 6.1 was used to quantitate the signal from each spot. The microarray data is included in Additional files 4,5,6.

### Microarray data analysis

The microarray data were managed using R-software [39] and Bioconductor package *marray*[43]. The microarray raw signals were processed as described previously [41]. Briefly, after local background subtraction, the control channel values were multiplied by the ratio of medians of probe channel and control channel. Next, negative values were removed and probe channel signals were adjusted as *L*_i_^'^ = *L*_i_log(*L*_i_/*C*_i_), where *L*_i_ is the raw probe channel signal value at feature *i* and *C*i is the adjusted control channel signal value at feature *i*. Further normalisation in sensitivity tests with Arrayit microarrays was executed by dividing all signals by a control ligation probe signal. Alignment of probe sequences to template sequences was done in R using local pairwise alignment functions from package *Biostrings*[40]. The used nucleotide substitution matrix had match score of 1 and mismatch score of −2. The microarray data have been deposited to ArrayExpress with accession numbers E-MEXP-3539 (sensitivity tests), E-MEXP-3541 (reactor samples), E-MEXP-3538 (specificity tests).

### Quantitative PCR experiments

A TaqMan probe (5'-AGGAACATGTGGTTTA-3') was designed to hybridise to the same position as the corresponding microarray ligation probe (A123). The probe harbored a 5' VIC® reporter dye, a 3' non-fluorescent quencher and a MGB™ (minor groove binder). The PCR reaction mixture for amplification of the TaqMan probe target region contained 1X Genotyping Master Mix (Applied Biosystems, Foster City, CA, USA), 900 nM forward primer (5'-GAAAGCGATAAGTTATCCACCTGGG-3'), 900 nM reverse primer (5'-TTCGAGCCCGGGTAAGGTTCC-3'), 250 nM TaqMan probe and approximately 50 ng of environmental DNA in a final volume of 20 μl. The PCR program consisted of activation at 95 °C for 10 min and 40 cycles of denaturation at 95 °C for 30s and annealing/extension at 60 °C for 1 min. Each reaction had three replicates in the assay plate. The reaction was carried out in StepOnePlus realtime PCR instrument (Applied Biosystems, Foster City, CA, USA).

## Results and discussion

### Biogas production

Anaerobic codigestion of biowaste and sewage sludge was performed with organic loading rates from 1 to 10 kg of VS m^-3^ d^-1^ in in mesophilic (M1 and M2) and thermophilic (M3 and M4) conditions. In the steady state conditions, i.e. the biogas production is not changed over time due to the load increase but has reached a constant level, the biogas production at the load of 3 kg VS m^-3^ d^-1^ was 680 and 760 liters kg^-1^VS^-1^ in the mesophilic and thermophilic runs, respectively (Table 2). In both temperatures the specific biogas production was lower at the loads of 5–8 kgVS m^-3^d^-1^ than that with 3 kg VS m^-3^d^-1^load. The CH_4_ concentration varied between 61.7 -68% in the both runs. The amounts of trace gases, especially ethanol and ammonia, increased in the thermophilic conditions.

### Overview of microbial diversity in AD

Selected samples from the outfeed of meso- (M1 and M2) and thermophilic (M3 and M4) pilot AD reactors at the loading rates of 3 and 5–8 kg VS m^-3^d^-1^ were subjected to microbial diversity analysis using 454 rRNA gene amplicon deep sequencing. A total of 77 189 sequences out of 83 975 sequence reads were classified based on BLASTN results. The total number of sequence reads that passed quality check ranged from 2 000 in Bacteria to almost 17 000 in Fungi per sample (Table 3). Figure 2 summarises the most abundant archaeal, bacterial and fungal groups present in the samples. Rarefaction analysis (Additional file 1) revealed that the fungal diversity increased together with increasing loading rate and decreasing retention time during the experiment, and Chao1 and Ace [27,28] richness estimates supported this observation (Table 3). In Bacteria, the trend in rarefaction analysis was the opposite, thus declining during the digestion process. Richness estimates in the mesophilic process backed up this result whereas in the thermophilic conditions the numbers were contradictory (Table 3). In Archaea, the diversity decreased during the experiment in the mesophilic and increased in the thermophilic reactor (Table 3). Several studies have shown that mesophilic AD process carries more microbial diversity than thermophilic process and that temperature affects the community composition of microbial communities [6,44-49]. In this study, rarefaction analysis (Additional Figure 1), richness estimates and diversity indices (Table 3) indicated approximately equal diversity in both temperatures. However, at class and genus level more bacterial classes and genera and archaeal genera were found in the mesophilic reactor than in the thermophilic reactor. Based on UPGMA (Unweighted Pair Group Method with Arithmetic mean) clustering [50] (data not shown), the bacterial and archaeal communities were more similar between the mesophilic samples (M1 and M2) than the thermophilc samples (M3 and M4), suggesting that bacterial and archaeal communities in the study reactors were strongly driven by temperature. In contrast, the fungal communities became more pronounced during the digestion process: the M1 and M3 samples taken in the beginning of the experiment from different reactors were more similar to each other than to M2 and M4 samples, suggesting that organic loading rate is a more important factor in determining the fungal community structure than the process temperature. As the digester was a completely stirred tank reactor, the new feed material is constantly mixed with old material while the mixture is being washed out. The operating time span before sampling was over one HRT in samples M1 and M3 and slightly less one HRT in samples M2 and M4 (Table 1, Figure 1). Due to constant stirring, this difference is not likely to have a major effect on the reactor microbiota. The minimum HRT used in this study was 9–10 days which is approximately the same as the generation time of methanogens and other microbial groups and as such is sufficient for proper decomposition of organic material. The efficiency of the degradation was also illustrated by the fact that no accumulation of degradation intermediates, i.e. VFA, occurred.

### Bacterial diversity

The mesophilc (M1 and M2) and thermophilic (M3 and M4) samples contained in total 15 bacterial phyla. Most commonly found bacterial phyla included *BacteroidetesFirmicutes* and *Thermotogae*, constituting 47%, 24% and 9% of all bacterial sequence reads, respectively. The phylum *Bacteroidetes* was more abundant in the mesophilic reactor, and the bacterial classes of *FlavobacteriaSphingobacteria* and *Bacteroidia* were found solely from the mesophilic reactor. *Clostridia* and *Bacilli*, the two classes of *Firmicutes*, were detected in both reactors but were more prevalent in thermophilic conditions, and *Thermotogae* was detected exclusively in the thermophilic reactor. Different classes of *Proteobacteria* and *Actinobacteria* were found in thermophilic conditions in quite small numbers, but these groups were substantially more abundant in the mesophilic reactor. *SpirochaetesSynergistes* and *Verrucomicrobia* were present only in the mesophilic reactor. We also detected several bacterial phyla comprised merely of environmental clones including OP8, OP11, SR1 and TM7. Somewhat concordant results regarding the heterotrophic bacteria in anaerobic digestors have been published before [51-54]*.* Bacterial phyla *BacteroidetesFirmicutes* and *Thermotogae* are often found in both mesophilic and thermophilic AD processes which reflects their importance in degradation of complex organic compounds [6].

Bacterial genera frequently encountered in AD include *Spirochaeta* sp., *Clostridium* sp., *Propionibacterium* sp., *Thermotoga* sp., *Arthrobacter* sp. and *Bacillus* sp. [8]. In the present study, 7% of all bacterial sequence reads were classified to genus level. All in all, we identified a total of 19 bacterial genera. The most common bacterial genus was *Clostridium*, present in all samples but more abundant in the thermophilic reactor. Genus *Clostridium* contains species that are capable of anoxic digestion of cellulose and fermenting amino acids, and these bacteria are commonly found in different types of anaerobic digesters [55]. In several earlier studies members of order *Clostridiales* have been detected to represent a dominant fraction of bacterial communities in AD and these bacteria are recognised important in biogas production [56-58]. *Coprothermobacter* sp. and *Syntrophomonas* sp. were also relatively common, with *Coprothermobacter* found solely in thermophilic and *Syntrophomonas* in both reactors.

### Archaeal diversity

We were able to identify 89% of all archaeal reads at phylum level and 34% at genus level. All the Archaea classified at phylum level belonged to phylum *Euryarchaeota*. This is in agreement with other descriptions of archaeal composition of anaerobic sludge where *Euryarchaeota* clearly dominate over *Crenarchaeota*, and orders *Methanosarcinales* and *Methanomicrobiales* are known to represent an eminent proportion of the Archaea present [59]. The two identified methanogenic classes were *Methanobacteria* and *Methanomicrobia*. These methanogens were found at both temperatures, although *Methanobacteria* were more prevalent in the thermophilic conditions (M3 and M4) than in the mesophilic conditions (M1 and M2). These classes represent typical archaeal constituents in methanogenic AD systems [54]. We identified also six different archaeal genera in our dataset based on BLAST against *nr*/*nt* database. *Methanosarcina* was very abundant, and slightly more common in the mesophilic process. *MethanobrevibacterMethanosphaeraMethanospirillum* and *Methanosphaerula* were abundant in mesophilic digestor (M1 and M2), while *Methanobacterium* was detected merely in thermohilic digestor (M3 and M4). In agreement with our study, Goberna and co-workers also found an increase of Methanobacteria in thermophilic AD [60]. Several studies have shown that *Methanosarcina* sp., *Methanococcus* sp. *Methanoculleus* sp., *Methanomethylovorans* sp. and *Methanobacterium* are typically found in anaerobic digesters [4,6,8-11].

### Fungal diversity

We identified 85% of the fungal sequences at phylum level and 44% at genus level. The Fungi detected in our study belonged to two phyla, *Ascomycota* and *Basidiomycota*. The sequence reads assigned to *Ascomycota* represented almost 99% of the fungal sequences and consequently, *Basidiomycota* constituted about 1% of the fungal reads. *Saccharomycetes* and *Eurotiomycetes* were the most abundant fungal classes in the whole dataset, constituting 58% and 12% of the fungal sequence reads, respectively. These classes were found in both temperatures, with *Saccharomycetes* being more abundant in the thermophilic digestor (M3 and M4) and *Eurotiomycetes* in the mesophilic digestor (M1 and M2) (Figure 2). A total of 33 fungal genera were detected. By far the most abundant was *Candida,* found in both processes at both samplings, but especially prevalently in the thermophilic reactor. The second most common fungal genus, *Penicillium*, was present in all samples but notably more thriving in the mesophilic reactor where it constituted the majority of all fungal sequence reads. The third most common fungus *Mucor* was found in all samples as well, but it seemed to prefer elevated thermophilic temperatures. In fact, several fungal groups, like *ZygorhynchusCladosporium* and *Pseudeurotium* were found solely in the thermophilic conditions, whereas for example *RhizomucorGeotrichum* and *Trichosporon* were found exclusively in the mesophilic reactor. The relative abundance of fungal groups like *PichiaSaccharomycesAspergillusMucor* and *Candida* increased during the digestion process, indicating that these fungal groups not only tolerate the conditions in the reactors but may actually benefit from them. *Pichia* and *Candida* are also associated in aerobic digestion [61]. Schnürer and Schnürer [12] recently studied fungal survival in anaerobic digestion of household waste and found out that mesophilic temperature did not reduce the amount of culturable fungal colony forming units in the waste, and that thermophilic conditions caused only a slight decrease in the number of fungal viable cells. This phenomenon was not detected in our study, but actually the thermophilic digestor (M3 and M4) contained more fungal diversity in both samplings compared to the mesophilic digestor (M1 and M2, Figure. 2). The majority of Fungi are aerobic, but a wide range of them are able to grow in low oxygen conditions. There are also fungi that can survive and grow in anaerobic conditions if an appropriate nutrient source is available. The fungal genera *CandidaMucorPenicilliumSaccharomyces* and *Trichoderma*, detected in our study, are facultative anaerobes and as such capable of degrading organic material in anoxic environment [62-64]. Thus, these groups can potentially not only survive the anaerobic conditions but also actively contribute to the process by decomposing more complex organic compounds such as lignin and cellulose in the beginning of the degradation.

### Functional validation of the microarray probes

Microarray as a high-throughput platform has the potential for routine microbial analysis of environmental samples [65-67], although detection accuracy of oligomeric probes targeting phylogenetic marker gene may present a challenge in analysing complex communities consisting of a large number of closely related genomes [16]. Assaying the microbial composition in the AD process would be valuable for in-process monitoring of the microbial content and confirming hygienisation of the end product. To that end, we applied ligation probes that circularize upon target recognition (“padlock probes”) and are subsequently amplified and hybridised on microarray by unique tag sequences (Figure. 3). In principle, the method enables detection from unamplified source material and has been previously successfully used for plant pathogen detection on qPCR [68] and microarray platforms [69] as well as for gene variant analysis [70,71]. However, to our knowledge, this type of technique has not been applied to profiling complex microbial communities to date.

**Figure 4:**
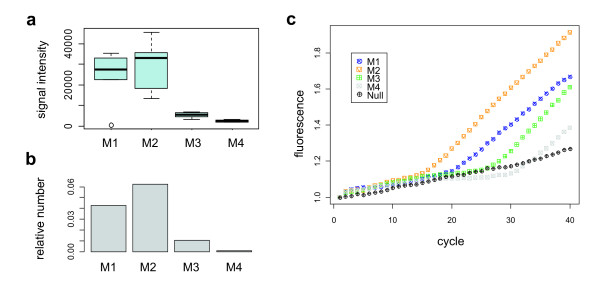
**Comparison of sequencing, microarray and qPCR.** Performance of probe A123 on samples M1, M2, M3 and M4. (**a**) Relative abundance of sequencing reads corresponding to microarray probe A123 bacterial target groups, (**b**) microarray signal intensities and (**c**) TaqMan assay using the same probe sequence.

Here, we tested a set of padlock probes to evaluate the potential of the method for AD process monitoring and more generally for microbial community analysis (Figure 4). In order to establish the functionality and target sequence specificity of the probes, we used 10 fmol of probe-specific synthetic dsDNA oligos as templates for the probe pool in ligation reactions. Signals from the subset of probes corresponding to the templates present in each pool could be clearly distinguished from signals from the rest of the probes (Additional file 4), suggesting a good target sequence specificity. However, the signal intensities of different probes varied considerably at the constant 10 fmol template concentration, probably because of random variability of PCR [72] and sequence bias of ligation [73,74]. Approximately 10% of the probes were not functional despite their perfect alignment to template. Six probes were non-specific giving false positive signals, despite that they did not have good alignment to any of the templates. To estimate the amount of detectable template, we tested template pools each containing 24 templates, at four different concentrations each. The probe signal intensities correlated with concentration (Additional file 5) with the highest concentration (1 fmol/μl/template) giving the highest signals while at the lowest concentration (0.001 fmol/μl/template) practically none of the probes produced detectable signals. Almost all of the probes had consistently lower signals with lower concentrations and the majority of probes were still detectable at 0.01 fmol/μl/template concentration, suggesting that the method may be used for semiquantitative assaying over at least three orders of magnitude.

### Microarray analysis of the AD samples

**Figure 5:**
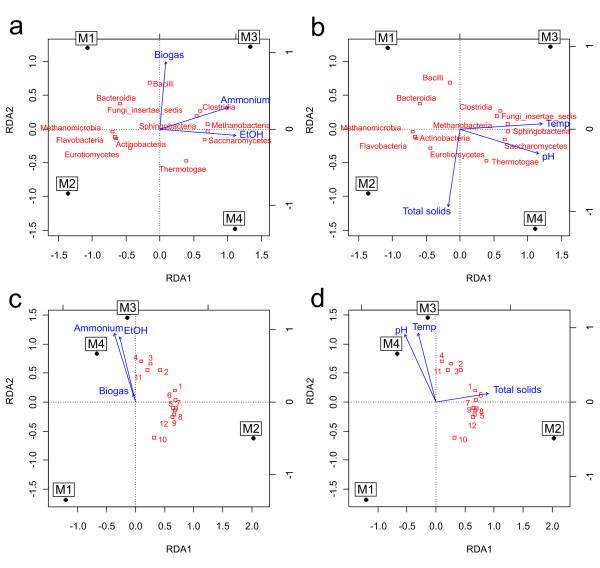
**Ordination of microbial composition together with physical and chemical parameters of AD samples.** Redundancy analysis (RDA) was used to explore the main trends in the data. The canonical axes represent principal components. Sample (M1-M4) locations relative to each other indicate their similarity in the ordination space. Red squares indicate microbial groups in sequence data (**a** and **b**) and probes in microarray data (**c** and **d**), with the numbers indicating the microarray probes listed in the Additional file 2. Only the most abundant groups or strongest probe signals were included in the analysis. Blue arrows indicate the physical and chemical parameters used as constraining variables in the analysis (from Tables 1 and 2). The length and position of an arrow illustrates its significance on the canonical axes.

To evaluate the microarray’s capability in analysing the AD samples, we performed ligation reactions using about 200 ng of non-amplified sample DNA as template for the probe pool. The microarray signals from the mesophilic samples M1 and M2 and the thermophilic samples M3 and M4 grouped separately and along the gradients of physical and chemical parameters in a similar way as with sequencing data (Figure 5) in redundancy analysis [16]. This suggests that our microarray had the ability to monitor changes in the microbial community structure in response to conditions of the digestor, an important aspect of in-process monitoring of AD status. However, while the grouping with M1 and M2 was comparable to sequencing data, M3 and M4 clustered less clearly separately showing that the microarray was not as accurate in classifying samples as deep sequencing with regard to process loading rate. The reason for this could be that most of the microarray probes did not show detectable signals. The probes were initially designed to match certain phylotypes or phylotype-level OTUs (97% read sequence similarity), but as these typically corresponded to relatively few sequences in the sample material, the target sequence abundances were likely to be below detection limit of the method. Also, specific microarray probes could not always be designed merely on the basis of trimmed 454 sequence reads due to their limited length of 150 nt, which necessitated us to retrieve full-length rRNA genes matching to OTUs from the NCBI nucleotide database. The closest matching gene to an OTU was typically only 94% similar, leaving considerable uncertainty regarding the estimated target specificity of the probes in the context of the AD sample DNA. Probe sequence alignments against the most abundant full-length database rRNA genes identified in the samples showed that many of the probes indeed did not have good matches. As expected under the probe-target sequence mismatch hypothesis, the probes that could be aligned with mismatches to the database rRNA genes were less accurate (Additional file 6) than 100% matching probes. Since the probes in the initial specificity tests responded highly accurately to their cognate target oligo pools, it is reasonable to assume that at least some missing signals are explained by unknown sequence differences in the rRNA genes. Secondary structures inherent to rRNA sequences are one possible contributor to probe target recognition [75] as well. However, we found complementarity within the probe pool only between two sequences (data not shown), but this does not completely rule out the possibility of dimerisation between other probes too, as alignment cannot fully explain oligo hybridisation behaviour. However, with 100% match to target sequences the signals were more consistent. Figure 4 shows microarray signals of a probe matching to several full length rRNA genes of uncultured bacterial groups, and corresponding relative number of 454 reads of these targets. The signals correlated with read number and TaqMan RT-qPCR signals obtained using the same probe sequence, thus verifying the microarray results. This proof of principle data suggests that the microarray method is capable of semiquantitative assaying of target microbial groups, provided the target sequences constitute at least 1% of total DNA in the sample as measured by amplicon sequence reads. Furthermore, the results show that sensitivity of the padlock method is clearly better compared to the traditional ligation detection reaction (LDR), which requires PCR amplification of the target sequences first, and is not able to detect targets directly from source DNA [66].

## Conclusions

Our results show that both the mesophilic and thermophilic AD process contain a prominent fungal community that survives and grows in anoxic conditions. This suggests that Fungi may metabolise organic nutrients for subsequent use by archaeal and bacterial methanogenic groups, thus contributing to the digesting process and biogas production. The microarray proof of principle testing showed the capability of the technique to profile the microbial composition of AD samples. According to our results, the microarray method is capable of semiquantitative analysis of AD process when comprehensive sequence information is available to support probe design. We expect future metagenomic sequencing of the total genomic content in these environments to enable more accurate probe design and, together with RNA sequencing, to help determining the ecology and metabolic functions of various fungal and other microbial groups present in the AD community.

## Competing interests

The authors declare that they have no competing interests.

## Authors’ contributions

MK, MR and JMK conceived the experimental design on anaerobic digestion. PA, LP, JH, JR and KK conceived the microarray and sequencing experiments. MK provided expertise on physical and chemical processes of digestion. JR performed the microarray and qPCR experiments and analysed the data. KK performed the PCRs for amplicon sequencing and analysed the sequence data. JR and KK did the RDA analysis and drafted the manuscript. All authors contributed to writing the manuscript and read and approved the final version.

## Supplementary Material

Additional file 1 **Figure of rarefaction curves of Archaea, Bacteria and Fungi in samples M1-M4.** (675 KB, PDF) (PDF 674 kb) Click here for file

Additional file 2 **Sequences of ligation probes.** Table containing the probe sequences and target Genbank accession numbers. (39 KB, XLS) (XLS 39 kb) Click here for file

Additional file 3 **Sequences of templates used in microarray specificity tests.** (40 KB, XLS) (XLS 39 kb) Click here for file

Additional file 4 **Microarray signals of specificity tests.** Boxplots of signals of each probe in response to artificial target template pools and alignment scores to sequences in the target pool. (273 KB, PDF) (PDF 273 kb) Click here for file

Additional file 5 **Microarray signals of sensitivity tests.** Figures showing microarray signals of different concentrations of synthetic template oligos. (47 KB, PDF) (PDF 47 kb) Click here for file

Additional file 6 **Example of microarray signals of mismatching probes**. Figures showing comparison of microarray signals and sequencing read numbers of two probes aligning with mismatches to groups present in samples M1-M4. (32 KB, PDF) (PDF 32 kb) Click here for file
