# Supplementary material for: Molecular analysis of meso- and thermophilic microbiota associated with anaerobic biowaste degradation
Source: BMC Microbiol. 2012 Jun 22;12:121. doi: 10.1186/1471-2180-12-121 (PMC3408363; doi:10.1186/1471-2180-12-121)
Supplement: Additional file 1 — Figure of rarefaction curves of Archaea, Bacteria and Fungi in samples M1-M4. (675 KB, PDF) (PDF 674 kb) [file 1471-2180-12-121-S1.pdf]

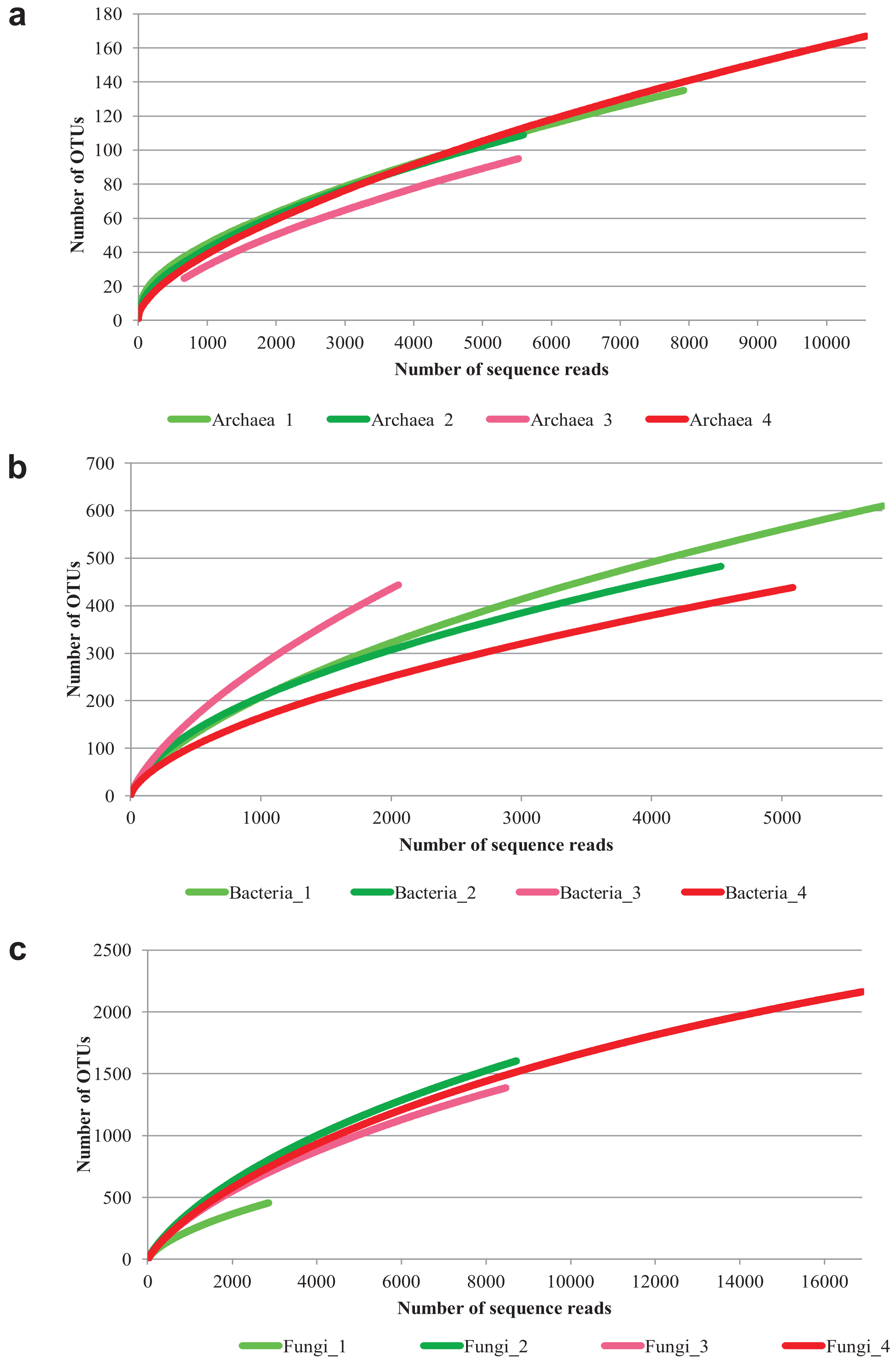

Additional file 1. Rarefaction curves representing the relation between the number of sequence reads and the number of operational taxonomic units (97% similarity). a) Archaea, b) Bacteria and c) Fungi.
