# Supplementary material for: Molecular analysis of meso- and thermophilic microbiota associated with anaerobic biowaste degradation
Source: BMC Microbiol. 2012 Jun 22;12:121. doi: 10.1186/1471-2180-12-121 (PMC3408363; doi:10.1186/1471-2180-12-121)
Supplement: Additional file 4 — Microarray signals of specificity tests. Boxplots of signals of each probe in response to artificial target template pools and alignment scores to sequences in the target pool. (273 KB, PDF) (PDF 273 kb) [file 1471-2180-12-121-S4.pdf]

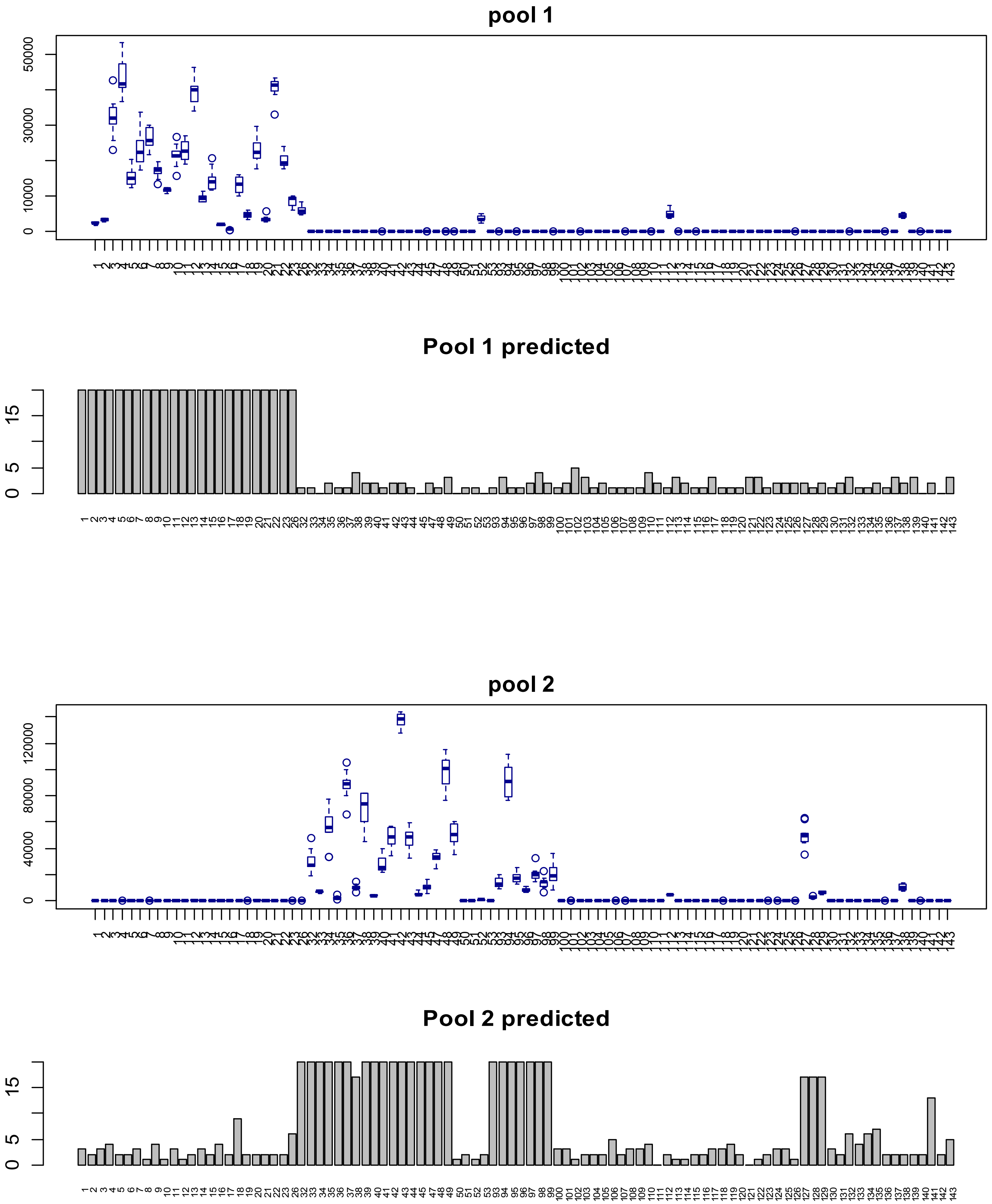

Additional file 4. Observed and predicted microarray probe signals from 10 fmol synthetic template pools. (Continues on next page.)

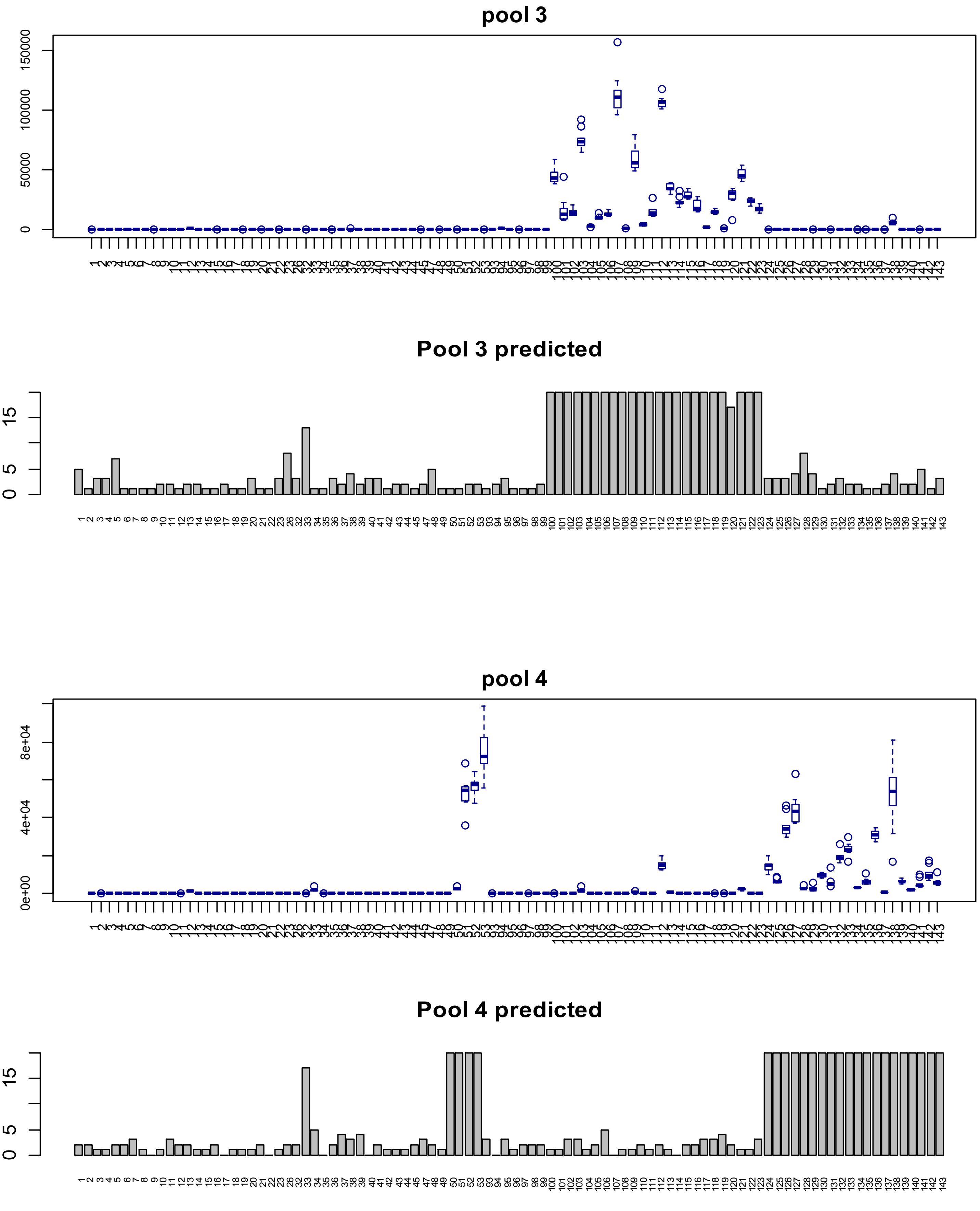

Additional file 4. (Continued from previous page.)
