# Supplementary material for: Molecular analysis of meso- and thermophilic microbiota associated with anaerobic biowaste degradation
Source: BMC Microbiol. 2012 Jun 22;12:121. doi: 10.1186/1471-2180-12-121 (PMC3408363; doi:10.1186/1471-2180-12-121)
Supplement: Additional file 5 — Microarray signals of sensitivity tests. Figures showing microarray signals of different concentrations of synthetic template oligos. (47 KB, PDF) (PDF 47 kb) [file 1471-2180-12-121-S5.pdf]

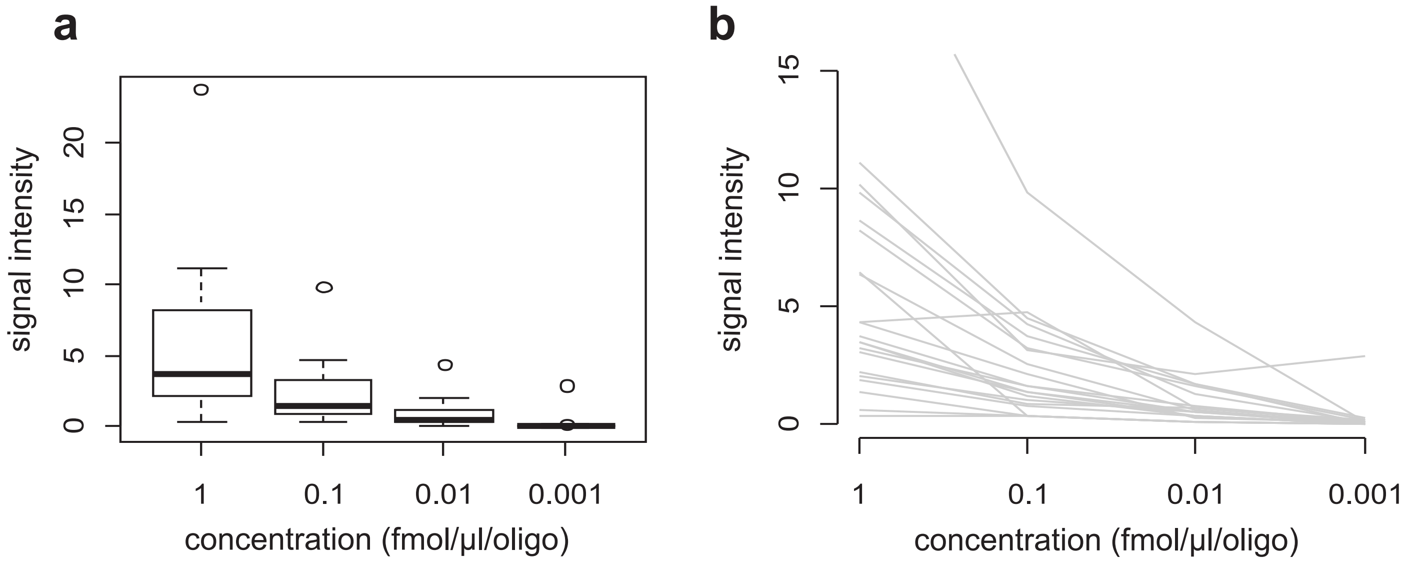

Additional file 5. Microarray signals of different concentrations of synthetic template oligos. (a) Boxplots showing the distribution of signals in each concentration and (b) line plots showing mean signals of individual probes.
