# Supplementary material for: Molecular analysis of meso- and thermophilic microbiota associated with anaerobic biowaste degradation
Source: BMC Microbiol. 2012 Jun 22;12:121. doi: 10.1186/1471-2180-12-121 (PMC3408363; doi:10.1186/1471-2180-12-121)
Supplement: Additional file 6 — Example of microarray signals of mismatching probes. Figures showing comparison of microarray signals and sequencing read numbers of two probes aligning with mismatches to groups present in samples M1-M4. (32 KB, PDF) (PDF 32 kb) [file 1471-2180-12-121-S6.pdf]

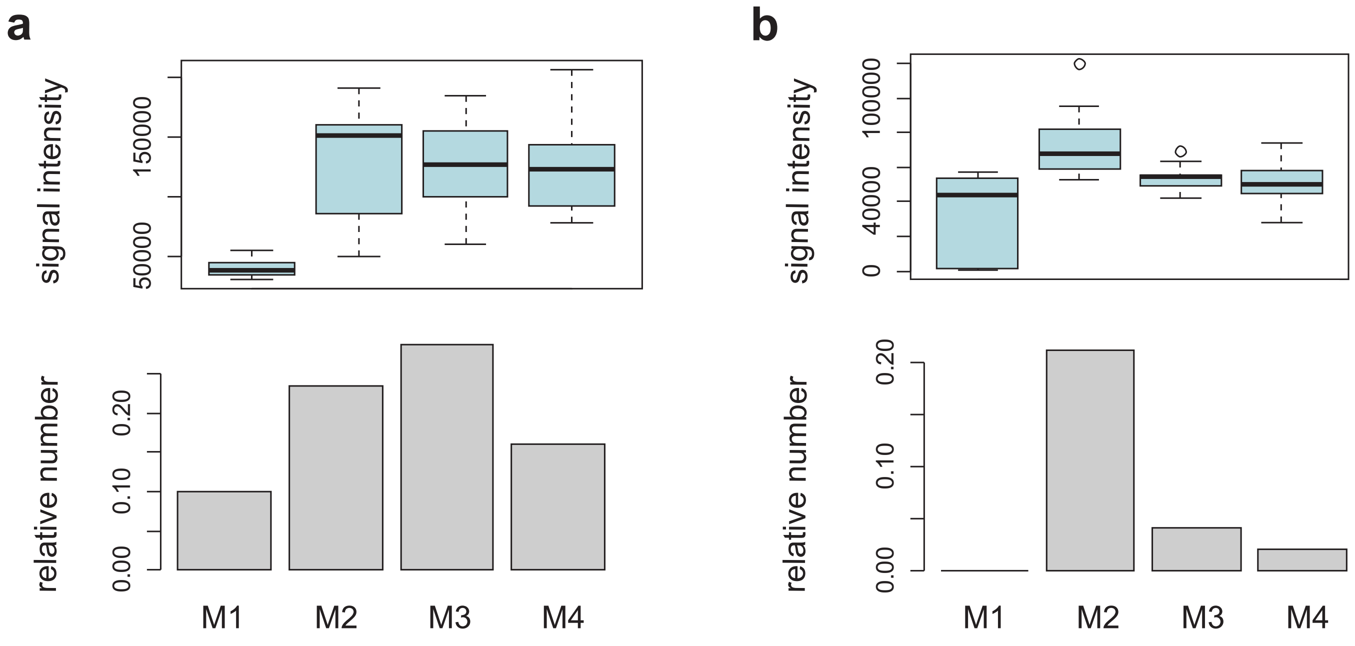

Additional file 6. Microarray signals of probes aligning with mismatches to full-length rRNA gene sequences present in the samples M1-4 (upper panels). In lower panels, relative 454 read numbers of corresponding sequences.
